# Precision Management of Complex Coronary Lesions: Drug‐Coated Balloons and Computational Cardiology at the Forefront of Nanotechnology

**DOI:** 10.1002/clc.70045

**Published:** 2024-11-08

**Authors:** Yashendra Sethi, Inderbir Padda, Sneha Annie Sebastian, Arsalan Moinuddin, Talha Bin Emran, Sunny Goel, Gurpreet Johal

**Affiliations:** ^1^ Department of Medicine Government Doon Medical College Dehradun India; ^2^ PearResearch Dehradun India; ^3^ Department of Medicine Richmond University Medical Center Staten Island New York USA; ^4^ Department of Medicine Azeezia Medical College Kollam Kerala India; ^5^ School of Sport and Exercise University of Gloucestershire Gloucester UK; ^6^ Department of Pharmacy Faculty of Allied Health Sciences, Daffodil International University Dhaka Bangladesh; ^7^ Department of Cardiology Icahn School of Medicine at Mount Sinai Hospital New York USA; ^8^ Department of Cardiology University of Washington, Valley Medical Center Seattle Washington USA

Dear Editor,

Complex coronary lesions present formidable challenges in interventional cardiology, necessitating innovative approaches for effective management. From myocardial bridging (MB) to ostial lesions and bifurcations, each poses unique anatomical and physiological hurdles. Despite advancements in coronary interventions, addressing these lesions remains a clinical conundrum owing to their diverse characteristics and associated complications. Traditionally, drug‐eluting stents (DES) are the primary choice for treating coronary artery stenosis, including lesions with myocardial bridges; however, such stents may exacerbate potential risks, including major adverse cardiac events, in‐stent restenosis, and postimplantation stent fracture; further compounded by prolonged dual antiplatelet therapy and its associated bleeding risks.

Stemmed from the theorized concept of “intervention without implantation” – drug‐coated balloons (DCB)—provide a simple yet pivotal alternative to DES (Figure 1). Employing semi‐compliant balloons loaded with antiproliferative drugs, DCBs penetrate into the local vessel wall, inhibiting intimal hyperplasia and promoting long‐term vessel patency. Previous studies like those by Xu et al. and Jeger Rv et al. have demonstrated their safety and efficacy in various coronary artery conditions, such as in‐stent restenosis, bifurcation lesions, small‐sized vessels, considerable lesion lengths [> 50 mm], high bleeding risk patients, de‐novo lesions, and patients planned for major surgery, e.g., coronary artery bypass graft [1, 2]. Table 1 summarizes the most recent (last 5 years) and relevant literature employing DCBs in complex coronary lesions. However, the considerations below are essential to understanding the underlying challenges and why their application has not gained traction.

**Figure 1:**
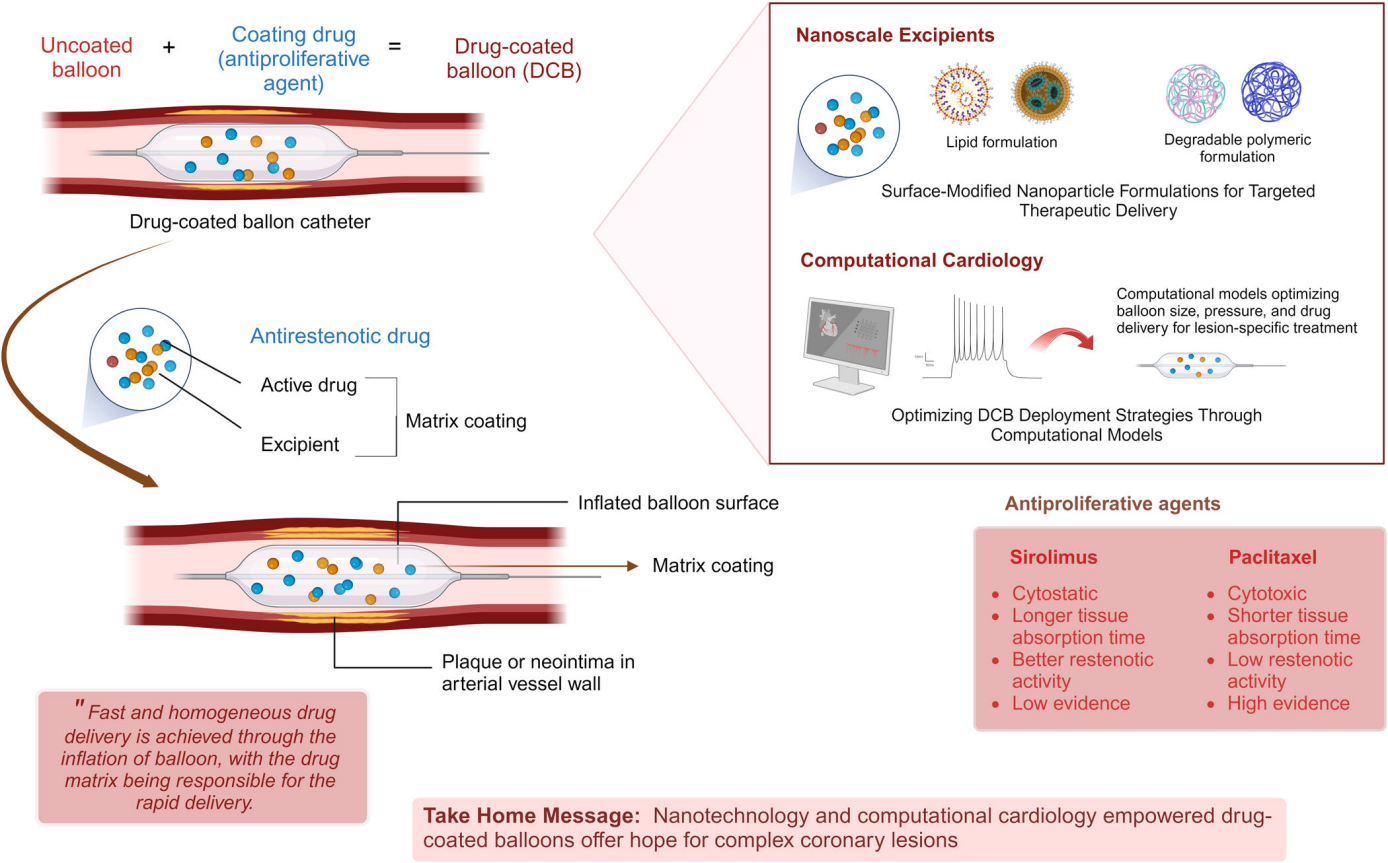
Integration of nanoscale excipients and computational models in utilizing drug‐coated balloons (DCB) for complex coronary lesions. This schematic illustrates the synergy between nanotechnology‐based additives and computational cardiology in optimizing the efficacy and precision of DCBs for complicated coronary lesions.

**Table 1:** Outcomes of various studies reporting the use of DCBs in complex coronary lesions.

References	Population	Outcome with use of DCB	Challenges faced/limitations	Other comments	PMID
Joh et al. [3]	Complex coronary lesions	Comparable risk of Target Vessel Failure between DCB and DES in complex lesions.	Further studies needed to confirm long‐term efficacy of DCB in broader complex lesion populations.	DCB might be a suitable alternative to DES in complex coronary lesions.	39101114
Korjian et al. [4]	Bifurcation	Improved long‐term vessel patency compared to conventional angioplasty; comparable to DES.	Need for further studies to confirm broader applications and efficacy.	DCBs may reduce the necessity for prolonged antiplatelet therapy in high‐risk patients.	38771909
Pan et al. [5]	Ostial lesion	Lower target lesion revascularization (4.90% DCB vs. 16.33% DES); lower MACE rate.	Need for randomized trials to validate findings; potential bias in retrospective design.	Suggests DCB as a viable alternative for managing ostial lesions.	37970224
Lu et al. [6]	Ostial lesion	DCB‐only showed less late lumen loss compared to hybrid strategy; similar safety and efficacy profiles.	Small sample size; need for further validation of findings through larger studies.	DCB‐only may be effective and technically easier than hybrid approaches.	36247262
Felbel et al. [7]	Small‐vessel CAD; Distal vessel segments	Comparable target lesion revascularization rates; lower all‐cause mortality with DCB compared to DES (1% vs. 3%).	Variability in study designs and patient characteristics across included studies; meta‐analysis limitations.	Supports the use of DCB as a promising alternative in managing ostial lesions effectively without stenting.	37671137
Shen et al. [8]	Myocardial Bridges	Successful treatment of atherosclerosis in myocardial bridging; no residual stenosis observed at follow‐up.	Clinical challenges in treating lesions due to physical compression during systole.	Highlights the role of intravascular imaging in assessing myocardial bridging severity	33778455
Zhang et al. [9]	Bifurcation	Similar safety profiles; higher incidence of coronary dissection in DCB group noted, but clinical outcomes comparable to DES.	Increased risk of delayed coronary dissection post‐DCB treatment; limited generalizability due to single‐center study design.	Emphasizes careful monitoring for complications associated with DCB use in bifurcations.	32842271

Of late, MBs have garnered significant attention for their association with acute coronary syndromes, coronary artery spasms, ventricular arrhythmias, and sudden cardiac death. Typically, MB denotes a congenital variation wherein a segment of a coronary artery traverses through the myocardium rather than taking its classic epicardial route. Given they are commonly hitched with atherosclerosis of proximal coronary arteries, DCBs offer a promising avenue; however, their precise delivery and long‐term efficacy are still to be determined [2, 8]. Recent reports have indicated DCBs as a potential treatment for atherosclerosis in the myocardial bridging segment, highlighting the benefits of the “leave nothing behind” strategy [10].

Ostial lesion analogs (OLAs): aorto‐ostial lesions, non‐aorto‐ostial lesions, and branch‐ostial lesions are typically found close to the ostium (≤ 3 mm) of the coronary artery. They are characterized by a rigid fibrotic texture (with pronounced sclerosis) which significantly enhances its propensity to recoil. Lesions at coronary ostia present unique challenges due to their anatomical location and hemodynamic implications. Although atherosclerosis is touted as the primary cause of OLAs, secondary lesions sometimes occur and are often associated with syphilitic vasculitis or aortic dissection. Lesions in aorto‐ostial regions (Medina classification 001 or 010) display increased elastic recoil, with post‐balloon dilation, raising risks of procedural failure, and possibly restenosis. Whilst stents counter elastic recoil, their misplacement can still lead to incomplete ostium coverage, increasing the chances of recurrence. To some extent, DCBs can provide a non‐implantable intervention option, partly via targeted delivery (to inhibit intimal hyperplasia) and by promoting long‐term vessel healing; nonetheless, the propensity to recoil and prolonged drug delivery still presents a challenge [2, 8, 9, 10, 11]. Employing the DCB strategy alone or in combination with the hybrid strategy has proven to be both safe and effective for the treatment of de novo ostial LAD/LCx lesions. This approach is characterized by a low technical threshold and a high success rate [5].

Coronary bifurcation lesions pose technical challenges (like carina shift, side branch closure, and geographical miss) during the intervention; DCBs offer a simplistic approach of injecting antiproliferative drugs directly to the site of the lesion, subsequently minimizing the need for complex stent placement. This significantly mitigates the risk of common stent‐related complications; for example, restenosis and thrombosis. Specifically, the provisional side branch (SB) stenting strategy, commonly used for bifurcation lesions, often has suboptimal outcomes. Indeed, a hybrid approach, namely, combining a DES in the main branch and a DCB in the ancillary SB, appears safe and effective, with possibly fewer complications and satisfactory midterm results [1, 2].

DCBs unquestionably offer a distinct mechanical advantage over stent‐based technologies by delivering drugs uniformly to the vessel wall, with both paclitaxel and sirolimus showing promising efficacy. While paclitaxel tends to localize predominantly in the subintimal space and adventitia, sirolimus exhibits slow absorption and has widespread distribution throughout the artery, posing challenges in maintaining adequate drug permeation. Finally, innovative approaches such as crystalline coatings, micro‐reservoir or nanotechnology for localized drug administration via balloons, and nanoscale biomolecular therapeutics are explored to address these challenges. Integrating DCBs with nanotechnology‐based therapeutics has supported the premise of inhibiting restenosis and reducing complications particularly for complex lesions like MBs, OLAs, and bifurcations, where precise intervention is crucial [2, 5, 8, 10, 11, 12].

More recently, the evolution of computational cardiology has circumvented some challenges encountered by both stents and DCBs. For example, the accurate blood flow patterns, shear stress distribution, and mechanical behavior within the vessel can be predicted by computational fluid dynamics simulations and finite element analysis. The simulation models and the computational models posit clinicians to optimize DCB deployment strategies, which encompass balloon size, inflation pressure, and drug delivery kinetics, specifically tailored to each lesion's anatomical and pathological features. Additionally, parallel imaging techniques provide valuable data by capturing phase‐locked images (with high‐resolution vessel geometry descriptions) to optimize DCB deployment strategies. This information further undergoes scrutiny with classical imaging techniques to produce corresponding wall surfaces. For example, high‐resolution MRI and automated detection techniques have been used to construct precise segmentations on stenosed carotid bifurcations. This facilitates the creation of a high‐resolution 3D model through a 2D watershed transform, streamlining the extraction of lumen boundaries, which can be critical for guiding effective DCB interventions. A modern approach with enhanced essential tools can be the key to computationally empowered interventional cardiology [13].

Collectively, the current evidence supporting the use of DCBs for complex MBs is compelling. While specific studies hint at the viability of a hybrid approach, the resounding safety and efficacy of DCBs across diverse coronary artery conditions herald them as not just another tool but a transformative intervention strategy. Yet, amidst this fervor, a crucial caveat remains, that is—the imperative for further research and robust evidence to fully validate their utility across a spectrum of lesion types and patient demographics. As we continue to explore novel therapeutic avenues and await large‐scale studies and clinical trials on computational models—DCBs hold promise in revolutionizing the management of complex coronary lesions. Computationally empowered interventional cardiology has the potential to drive precision medicine and improve patient outcomes.

## Author Contributions

**Yashendra Sethi:** conceptualization, validation, writing–original draft, visualization, project administration, writing–review and editing. **Inderbir Padda, Sneha Annie Sebastian,** and **Talha Bin Emran:** writing–original draft, review and editing, illustration. **Arsalan Moinuddin** and **Sunny Goel:** validation, writing–original draft, review and editing, final approval of manuscript. **Gurpreet Johal:** supervision, conceptualization, validation, writing–original draft, review and editing, final approval of the manuscript.

## Ethics Statement

The authors have nothing to report.

### Tweet

New drug‐coated balloons offer hope for complex coronary lesions, promising safer interventions. #Cardiology #Innovation.

## Data Availability

Data sharing not applicable to this article as no datasets were generated or analyzed during the current study. All source documents can be provided by the corresponding author upon motivated request.
